# A Review of Thromboembolic Events in Hospitalized COVID-19 Patients

**DOI:** 10.21203/rs.3.rs-393440/v1

**Published:** 2021-04-05

**Authors:** Paul Fontelo, Mrigendra M Bastola, Zhaonian Zheng, Seo Hyon Baik

**Affiliations:** National Library of Medicine; National Library of Medicine; National Library of Medicine; National Library of Medicine

**Keywords:** thromboembolic disorders, COVID-19

## Abstract

**Context:**

A higher incidence of thromboembolic disorders in COVID-19 has been reported by many clinicians worldwide.

**Objective, Design and Data Sources:**

Selected studies found in PubMed that reported thromboembolic events were included for meta-analysis using weighted fixed and random effects. Data from 19 articles on cohort studies in patients diagnosed with COVID-19 and thromboembolic events, including thrombosis and embolism were included in this review.

**Results:**

The likelihood for developing thromboembolic disorders in hospitalized COVID-19 patients was 0.28 (95% CI 0.21–0.36).

**Conclusion:**

This study further validates the increased risk of VTE in COVID-19 patients when compared to healthy, non-hospitalized people, and hospitalized patients. These findings will be useful to researchers and medical practitioners caring for COVID-19 patients.

## Introduction

Some viral infections manifest clinically with hemorrhage and coagulation syndromes. These may run the spectrum of mild skin hemorrhages to disseminated intravascular coagulation. Dengue, endemic in the Caribbean and in Asia, may present as skin rashes and petechiae in its mild form, but may be also associated with hemorrhagic shock syndromes in severe cases. Viral hemorrhagic fevers, like Ebola, Marburg, Lassa fever, Rift Valley fever and Crimean Congo fever, named after geographic locations where they were first discovered or are most prevalent, trigger hemorrhages of varying degrees of severity, some associated with high morbidity and mortality. Some patients with cytomegalovirus and parvovirus B19 may develop clotting abnormalities, like thrombosis. Viral respiratory tract infections are known to increase the risk of deep venous thrombosis and possibly pulmonary embolism [1].

Reports on coronaviruses did not appear in the literature until the 1960’s. Early documented cases of coronaviruses (HcoV-OC43, HcoV-NL63, HcoV-229E, and HKU1) were reported to produce only mild upper respiratory infections in immunocompromised patients. In 2003, the sudden appearance of the highly pathogenic Severe Acute Respiratory Syndrome (SARS-CoV) in Asia spread to more than two dozen countries worldwide before disappearing in mid-2003. SARS was followed in 2012 by another highly pathogenic coronavirus, the Middle East Respiratory Syndrome coronavirus (MERS CoV) [2–5]. MERS, a zoonotic disease which spread mostly among Middle East countries including Saudi Arabia, Jordan, Qatar, Oman, Kuwait, and UAE, eventually reached Europe. Smaller outbreaks have occurred subsequently among healthcare workers, but it has been generally contained. Patients with severe MERS developed pneumonia and kidney failure with about 35% of patients dying of the disease [6]. The multi-country epidemics of SARS and MERS were associated with coagulation disorders. Severe SARS patients developed thrombocytopenia, disseminated intravascular coagulation (DIC), deep vein thrombosis (DVT) and pulmonary embolism (PE) [8–9], while MERS was associated with intracerebral hemorrhage and DIC [10–11].

SARS-CoV-2, the etiologic agent of COVID-19, is a highly infectious coronavirus responsible for the current global pandemic. As of March 29, 2021, it has claimed more than 2.7 million lives and infected 127 million people globally since it was first reported in December 2019 [12]. Although the mortality rate is lower than MERS or SARS, it is more infectious and highly contagious [13]. Vascular complications, such as stroke, thrombosis, and embolism, have accounted for many of the fatalities. COVID-19 infection has also been associated with hypercoagulability with development of ischemic changes, including gangrene of fingers and toes. Disseminated intravascular coagulopathy was found in Chinese patients [14].

Several factors lead to the hypercoagulability state in patients with severe cases of COVID-19: circulatory stasis from immobility (common to intensive care patients), acute inflammatory reaction overdrive with increases in acute phase proteins (e.g., fibrinogen, c-reactive protein) and elevated clotting factors, increased Von Willebrand Factor (vWF) activity, neutrophilia, and increase in Neutrophil Extracellular traps (NETs) [15]. Reports have also shown possible direct endothelial injury [16–17] and increased blood viscosity in COVID-19 patients that may further result in thrombogenesis [18]. In addition, the hypoxia found in severe COVID-19 can stimulate thrombosis not only by increasing blood viscosity, but also a hypoxia-inducible transcription factor-dependent signaling pathway [19]. Large-vessel stroke has been reported as a potential early presentation of COVID-19 patients [20]. All the elements of Virchow’s triad - hypercoagulability, stasis, and endothelial injury and dysfunction - can present in COVID-19 patients.

Several reports of the thromboembolic consequences of COVID-19 have recently been published. The aim of this study is to determine the incidence of thromboembolic events in hospitalized COVID-19 patients.

## Methods

**Figure 1:**
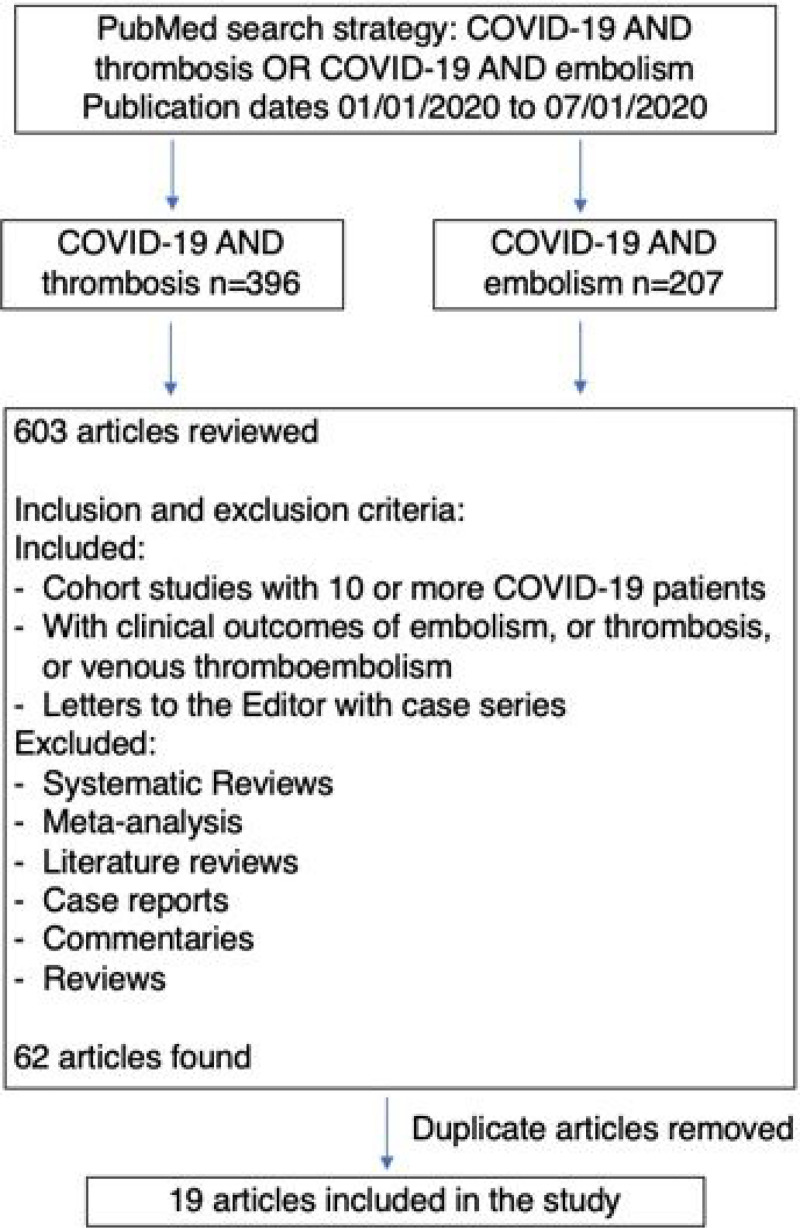
Flow Diagram of the Search Protocol

This study followed PRISMA guidelines for conducting meta-analysis [21]. PubMed searches were performed from July 6 to July 8, 2020 for articles published between January 1,2020 and July 1,2020. Searches were limited to PubMed because of the unprecedented increase in COVID-19 publications (already more than 35,000 publications from January 1,2020 to June 30, 2020). Using the search terms, “COVID-19 AND Thrombosis”, 396 articles were found, while the search for “COVID-19 AND Embolism” found 207 articles. Duplicate publications were deleted, and only independent research articles were included in the review. Letters, commentaries, opinions, perspectives and review articles, including systematic reviews and meta-analysis were excluded. However, research letters that included patient cohorts were included. Of the 62 articles found, 19 articles that had data on cohort studies in patients diagnosed with COVID-19 and vascular findings, including thrombosis, embolism, and endothelial injury, were included in this meta-analysis. The search strategy is summarized in Fig. 1. Table 1 lists the 19 selected studies with relevant information on period of study, mean age, sex, venous thromboembolic (VTE) effects and clinical outcomes for each study.

Table 1 shows the Attributes of the 19 Studies Included in the Meta-Analysis

Data were analyzed using StatDirect 3 (StatsDirect Ltd) and Rstudio, Version 1.2.5033 (RStudio, Inc). Proportions were transformed using the Freeman-Tukey double arcsine method [22] and were combined separately using an inverse-variance weighted fixed method and random effect method (DerSimonian-Laird estimator for Tau^2^) [23] and by the Jackson method for confidence interval of Tau^2^ and Tau [24]. While the inverse-variance weighted fixed method does not account variation across 19 studies, the random effect method does. Visualization for bias detection and assessment was plotted. Bias testing was performed using Begg-Mazumdar, Harbord and Egger tests.

## Results

**Figure 2:**
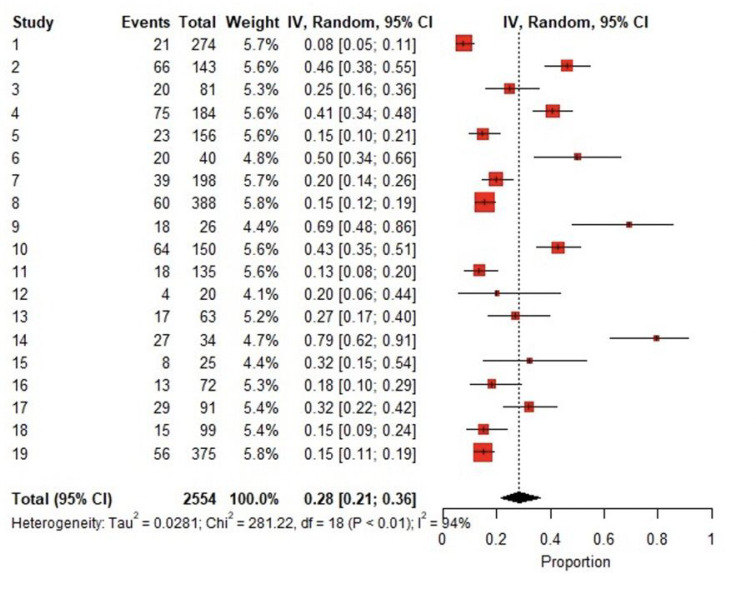
Forest Plot of the Analysis

The total pooled COVID-19 patient population was 2554. The forest plot of results of the analysis in Fig. 2 shows the likelihood (95% CIs) of thromboembolic events in this COVID-19 population. The pooled incidence rate of development of thromboembolic disorder was 0.28 (95% CI 0.21–0.36). Egger test with a P-value of 0.014 illustrates further significant publication the bias. A P-value less than 0.05 implies publication bias [25, 26].

Pooled proportion of VTE using the fixed effect method was 0.22, but the heterogeneity measure of studies (I^2^) was large, 93.6% (95% CI 91.3%−95.3%). A random effects analysis was used instead to generate the forest plot which gave an inverse variance value of 0.28 (95% CI 0.21–0.36). The pooled estimates of the odds ratios from the random effect meta-regression analyses for effect of four variables of interest on developing VTE (age, thromboprophylaxis, ICU admission and sex) were not significant.

## Discussion

Earlier reports have shown increased incidence of thromboembolic events in COVID-19 patients that is confirmed by this meta-analysis. The pooled incidence rate from the analysis of 19 studies indicates that about 28% (95% CI 21 % – 36%) of COVID-19 patients will develop venous thromboembolic events, a higher incidence than in the general population, hospitalized ICU and non-ICU patients. Two reports of cohort studies [27, 28] that included patient controls showed lower incidence of VTE in the control population, 5% and 10%, respectively, much lower than 28% found in COVID-19 patients in this review. In a study among county residents, Heit et al [29] found that the average annual incidence (adjusted by age and sex) of in-hospital VTE was 960.5 (95% CI, 795.1–1125.9) per 10,000. The incidence among non-hospitalized community residents was 7.1 (95% CI, 6.5–7.6) per 10,000 person-years or 100 times lower [29]. Among ICU patients, the cumulative incidence of VTE at 28 days, determined by weekly intervals was 4.45% (95% CI 2.55–7.71) [30].

Hospitalization increases the risk for VTE. In a review based on the 2003 Nationwide Inpatient Sample from the Healthcare Cost and Utilization Project (HCUP by the American College of Chest Physicians (ACCP) showed the risk for developing VTE among surgical patients classified as low, moderate, high, and very high were 44%, 15%, 24%, and 17% respectively [31]. Among medical patients, 51% (7.7 million) fit the American College of Chest Physicians (ACCP) VTE risk criteria. Even after discharged from the hospital, 31 % (12 million) patients continued to be at risk of VTE overall [31]. However, evidence seems to implicate infections with the SARS-CoV-2 with thromboembolic complications more than just hospitalization. In a study by Helms et al that compared 145 non-COVID-19 ARDS patients with 77 COVID-19 ARDS patients [32], they found that COVID-19 patients developed significantly more thromboembolic complications, mainly pulmonary embolisms (11.7 vs. 2.1%, p< 0.008). Another study by Poissy et al [33], compared 107 ICU COVID-19 patients with historical controls of influenza patients admitted to the same ICU in the previous year, and to another group of patients hospitalized with influenza. Their analysis showed more COVID-19 patients developed PE (20.6%), in contrast to PE rates of 6.1% and 7.5%, in the general ICU population and the influenza population, respectively [33].

ICU patients are predisposed to developing thromboembolism from all elements of Virchow’s triad. Two more papers in this review provide evidence that ICU patients with COVID-19 are at greater risk to VTE. Lodigiani, et al reported that in 388 COVID-19 patients, thromboembolic events occurred in 27.6% of ICU patients but only 6.6% general ward patients [27]. In another study by Middeldorp et al comparing 75 ICU and 123 ward patients with COVID-19, VTE occurred in 47% (35/75) of ICU patients [34]. Asymptomatic VTE was diagnosed in only 3% of ward patients [34]. A meta-regression of the entire study showed that ICU patients are 104% more likely to develop VTE although this was not significant (p = 0.165). Ward patients, who are likely to ambulate more, might be less prone to develop VTE.

Several societies and organizations have advanced recommendations, guidelines, and consensus statements regarding anticoagulation and COVID-19 patients. The American Society of Hematology (ASH) suggests using prophylactic-intensity over intermediate-intensity or therapeutic-intensity anticoagulation for patients with COVID-19–related critical illness who do not have suspected or confirmed VTE [35]. The American College of Cardiology also recommends that all patients hospitalized with COVID-19 receive pharmacologic VTE prophylaxis unless a specific contraindication (such as active bleeding) exists [36]. The NIH COVID-19 Treatment Guidelines states that “there are currently insufficient data to recommend either for or against the use of thrombolytics or higher than the prophylactic dose of anticoagulation for VTE prophylaxis in hospitalized COVID-19 patients outside of a clinical trial.” [37].

The entire population of 2554 patients were considered as a cohort of patients diagnosed with COVID-19 and the individual study effects of anticoagulation or specific anticoagulant use was not accounted for except to note that the majority of patients in this study (93.5%) were given some type of anticoagulant, variously described as prophylactic, intermediate, or therapeutic. Each study was weighted to account for its number of patients.

The differences between studies were large with a 94% I^2^ inconsistency value, therefore, a random effects model with pooled proportion (= 0.28) was adopted to account for the heterogeneity of the 19 studies reviewed. Also, between-study heterogeneity was large, and tests of bias were statistically significant indicating a “small sample” bias across the 19 studies.

In order to explore the association between patient characteristics and VTE development, a meta-regression was used. Since a fixed effects meta-regression model does not account for high heterogeneity across different studies, a random-effect meta-regression analysis was adopted although the meta-regression results for the effects of age, gender, thromboprophylaxis and ICU admission did not attain statistical significance. Possible intercorrelation between thromboprophylaxis and other variables could exist - not controlling for such intercorrelations could yield misleading information. For example, patients given thromboprophylaxis might have greater medical burdens that those without and thus they are more vulnerable to VTE.

### Limitations

The literature search was limited only to PubMed because of the unprecedented increase in COVID-19 publications. The 19 studies reviewed came from several countries and were very heterogeneous. Additionally, the effects of international variations in patient populations, testing strategies, thrombosis prophylactic measures, diagnostic test quality and availability, access to care and treatment strategies, as well as variability in outcome reporting for COVID-19, might also be a limitation. However, the adoption of the random effect approach instead of fixed effect approach might compensate for the diversity. These issues influence the reported diagnosed cases, casualties, and, in turn case-fatality rates. The incidence reported in this study might change as more cohort studies are reported and clinicians learn more about COVID-19 and its management. Publication bias brought about by publications analyzed in this review depend on a large extent on what their authors might consider as significant or perceive as important - these factors are beyond the control of this review. This study may have failed to include all relevant studies which might affect the estimated incidence. With COVID-19 now a worldwide pandemic, non-English publications may have also been missed (language bias). The large heterogeneity (I^2^ = 94%) also indicates a sample bias.

## Conclusion

This study provides more evidence that COVID-19 increases the risk of VTE. Although the majority of the reports did not have a control group, a comparison with historical groups of patients in the general community, hospitalized patients, and ICU patients showed a significant difference between the incidence of thromboembolism in COVID-19 patients. Vulnerable patients, such as the elderly, and those with other chronic comorbid conditions have greater risk of hospitalizations and, even critical care unit admissions, which will further predispose them to even greater risk of thromboembolism. The consensus among experts supports anticoagulation in all hospitalized COVID-19 patients. The findings of this study might be potentially useful to medical practitioners who care for COVID-19 patients who are at higher risk of developing thromboembolic events.

## Figures and Tables

**Table 1:** Attributes of the 19 Studies Included in the Meta-Analysis

Author	Study Period	Type of Study	# VTE/Total patients (%)	Mean age	Sex	Thromboprophylaxis (type/dose)	Clinical course
Stoneham SM, et al^38^	Mar 20-Apr 9, 2020 (20 days)	Case-control study	21/274 (8%)	VTE-positive 67 ± 12 years VTE-negative 65 ± 15 years	VTE-positive: Men 67% VTE-negative: Men 57%	3 patients given weight-based treatment with LMWH	Overall all-cause mortality rate 27.7%
Zhang L, et al^39^	Jan 29-Feb 29, 2020 (31 days)	Retrospective cohort study	66/143 (46%)	63 ± 14 years	Men 52% Women 48%	37.1% patients given DVT prophylaxis; 41.3% patients received LMWH after positive ultrasound studies for DVT	10.5% patients were admitted to the ICU. DVT patients > 65 years (66.7% vs 41.6%) and critically ill (65.2% vs 28.6%).
Cui S, et al^40^	Jan 30-Mar 22, 2020 (23 days)	Cohort study, risk analysis	20/81 (25%)	59.9 ± 14.1 years	Men 46% Women 54%	No preventive anticoagulant was administered	All admitted to ICU. 41% patients had chronic medical illness. D-dimer level was a good index for predicting VTE.
Klok FA, et al^41^	Mar 7-Apr 5, 2020 (29 days)	Prospective cohort study	75/184 (39%)	64 ± 12 years	Men 76% Women 24%	All patients received pharmacological thromboprophylaxis per local hospital	VTE patients at higher risk of all-cause death (HR 5.4). Anticoagulation lowers risk HR 0.29; all-cause death (HR 0.79, 95%CI 0.35–1.8).
Demelo-Rodríguez P, et al^42^	mid-April 2020	Prospective observational study	23/156 (15%)	68.1 ± 14.5 years	Men 65% Women 35%	All patients received standard doses of thromboprophylaxis, except 3 patients with high bleeding risk	Asymptomatic patients not in-ICU with COVID-19
Pavoni V, et al^43^	Feb 28-Apr 10, 2020 (11 days)	Retrospective, observational study	20/40 (50%)	61 ± 13 years	Men 60% Women 40%	All patients received thromboprophylaxis with low molecular weight heparin	DVT) in 6 patients (15%) and TBE 2 patients (5%); 12 patients (30%) had a catheter thrombosis
Middeldorp S, et al^34^	Mar 2-Apr 12, 2020 (41 days)	In-patient cohort study	39/198 (20%)	61 years	Men 66% Women 34%	Ward patients received thrombosis prophylaxis with nadroparin. ICU received a double dose of nadroparin	VTE 47% ICU patients, 3% of wards
Lodigiani C, et al^27^	Feb 13-Apr 10, 2020 (26 days)	Retrospective study	60/388 (21%)	66 (55–85) years	Men 80% Women 20%	All ICU patients received LMWH; general wards: prophylactic 41%, 21% intermediate-, 23% therapeutic dose.	Older patients dying during hospitalization (OR 1.10; 95%CI 1.07–1.13). VTE, 27.6% ICU, 6.6% general ward
Llitjos JF, et al^28^	Mar 19-Aprl 11, 2020 (23 days)	Retrospective cohort study		68 (51.5–74.5)	Men 77% Women 23%	31% treated with prophylactic dose, 69% with therapeutic dose	All ICU patients. 56% with VTE
Helms J, et al^32^	Mar 3-Mar 31, 2020 (28 days)	Multicenter prospective cohort		63 [53; 71] years	Men 81% Women 19%	70% prophylactic dose, 30% therapeutic dose	All ICU patients. PE16.7%. COVID-19 ARDS patients developed had more VTE (11.7 vs. 2.1%)
Koleilat I, et al^44^	Mar 1-Apr 10, 2020 (40 days)	Single center retrospective case-control study	18/26 (69%)	DVT positive – 59 years DVT negative – 64 years	Men 52% Women 48%	12/18 with chemical thromboprophylaxis; 2/18 therapeutic anticoagulation developed DVT	DVT 10.1% either SARS-CoV-2 negative or untested. More COVID-19 patients with DVT
Zerwes S, et al^45^	Apr 18-Apr 30, 2020 (12 days)	Prospective single center study	64/150 (43%)	Mean for all patients 67 years; COVID-19 patients 62 years, non-COVID-19 patients 69 years	No information	Anticoagulation: 9 prophylactic (6 COVID) 3 sub-therapeutic 5 therapeutic.	ICU patients: 20 COVID-19-positive patients compared with 20 non-COVID-19 patients. Elevated Ddimer levels.
Thomas W, et al^46^	Days of observation = 8 (range 1–28)	Observational study	17/63 (27%)	Estimated average age 61 years	Men 69% Women 31%	Prophylactic dalteparin adjusted for weight and renal function or unfractionated heparin	All ICU patients. At censor date: Still in ICU 44%; In ward or discharged 32%; Dead 16%
Nahum J, et al^47^	Mid-Mar to early Apr 2020 (21 days)	Prospective single center study	27/34 (79%)	62.2 ± 8.6 years	Men 78% Women 22%	All patients received anticoagulant prophylaxis at hospital admission	All in ICU. VTE 65% at admission, 79% 48 hrs after
Longchamp A, et al^48^	Marc 8-Apr 4, 2020	Retrospective review	8/25 (32%)	68 ± 11 years	Men 64% Women 36%	Therapeutic anticoagulation only in patients with VTE	Discharged 72% In hospital ICU 2% Dead 20%
Gervaise A, et al^49^	Mar 14-Apr 6, 2020 (23 days)	Retrospective review	13/72 (18%)	APE 74.4 years ± 15.0 non-APE 59.6 years ± 17.4	Men 75% Women 25%	Unknown	Discharged 38 (53%) In hospital 23 (32%) Dead 11 (15%)
Mestre-Gómez B, etal^50^	Mar 30-Apr 12, 2020 (13 days)	Retrospective review	29/91 (32%)	65 years (56–73)	Men 72% Women 28%	Most patients diagnosed with PE received LMWH, 79.3%	Discharged 82.7%; Still In hospital 13.8%; ICU 6.9%; Dead 3.4%
Inciardi RM, et al^51^	Mar 4, 2020-Mar 25, 2020 (21 days)	Prospective cohort study	15/99 (15%)	67 ± 12 years	Men 81%) Women 19%	Anticogulation not routinely given to patients in sinus rhythm	VTE higher in cardiac patients (23% vs. 6%) Mortality higher in cardiac patients (36% vs. 15%)
Soumagne T, etal^52^	Mar 10-Apr 12, 2020 (33 days)	Retrospective review	56/375 (15%)	With PE: 61.1 ± 9.1years Without PE: 63.5 ± 10.1 years	With PE: Men 84% Without PE: Men 76%	All patients given anticoagulation at preventive dose	Patients with PE vs. Pts without PE ICU mortality day 14: 16% vs. 26%) p = 0.13 ICU mortality day 28: 29% vs. 37%) p = 0.27 Extubated day 28: 49% vs. 68% p = 0.25

Abbreviations used in Table 1: Acute pulmonary embolism (APE); Deep vein thrombosis (DVT; Hazard Ratio (HR); Low molecular weight heparin (LMWH); Odds ratio (OR) Pulmonary embolism (PE); Sepsis-related Organ Failure Assessment (SOFA); Versus (vs)

## Abbreviations

COVID-19: Coronavirus Disease 2019
CI: Confidence Interval
VTE: Venous thromboembolism
ARDS: Acute Respiratory Distress Syndrome
